# Supplementary figures and images for: Altered auditory feature discrimination in a rat model of Fragile X Syndrome
Source: PLoS Biol. 2025 Jul 1;23(7):e3003248. doi: 10.1371/journal.pbio.3003248 (PMC12237272; doi:10.1371/journal.pbio.3003248)

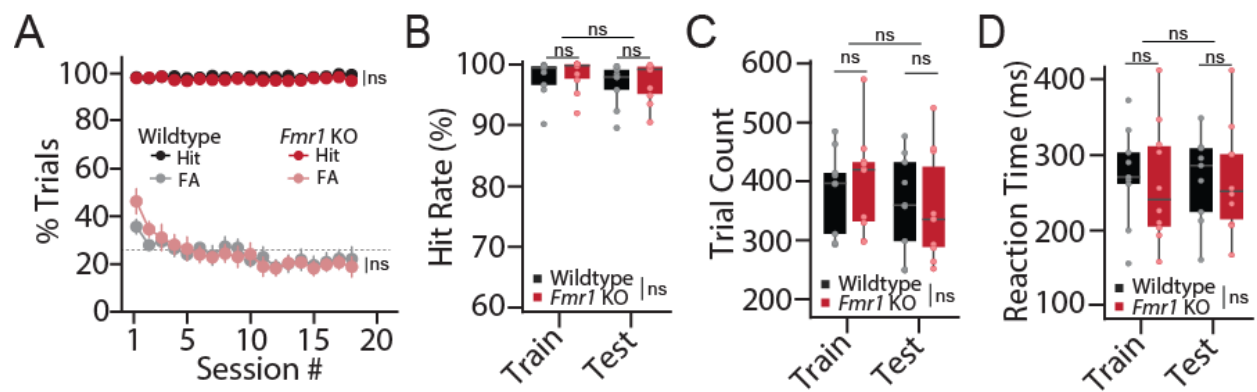

Supplement: S1 Fig — (A) Hit and false alarm (FA) rates over training sessions for 9 wildtype (black) and 10 Fmr1 KO (red) rats. No significant difference in hit rate (2-way ANOVA, genotype: df = 1, F = 1.031, p = 0.3284) or FA rate (2-way ANOVA, genotype: df = 1, F = 0.0004, p = 0.9828) was observed between wildtype and Fmr1 KO animals, demonstrating that both genotypes can learn and perform octave discrimination at comparable levels. (B) Comparison of average hit rate on the last two days of octave training (Train) and discrimination testing days (Test). There was no significant effect of task condition (Kruskal–Wallis: df = 1, p = 0.891) or genotype (Kruskal–Wallis: df = 1, p = 0.356) on hit rate. (C) Comparison of average trial count on the last two days of octave training (Train) and discrimination testing days (Test). There was no significant effect of task condition (2-way ANOVA: df = 1, F = 1.908, p = 0.159) or genotype (2-way ANOVA: df = 1, F = 0.031, p = 0.860) on trial count and no significant genotype~condition interaction (2-way ANOVA: df = 2, F = 0.198, p = 0.821). (D) Comparison of average reaction time on the last two days of octave training (Train) and discrimination testing days (Test). There was no significant effect of task condition (2-way ANOVA: df = 1, F = 0.506, p = 0.679) or genotype (2-way ANOVA: df = 1, F = 0.008, p = 0.930) on reaction time and no significant genotype~condition interaction (2-way ANOVA: df = 2, F = 0.069, p = 0.933). Box plots represent the median, 25th, and 75th percentiles. Whiskers represent the minimum and maximum values except for outliers. Boxplots dots represent individual animals. All other values are means ± SEM. ns = not significant. Source data for panels B–D are in S1 Data, S1 sheet. (PDF) [file pbio.3003248.s001.pdf]

A

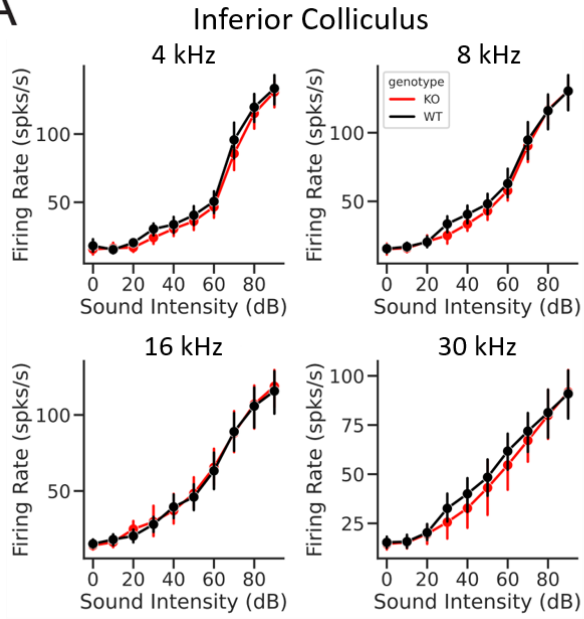

B

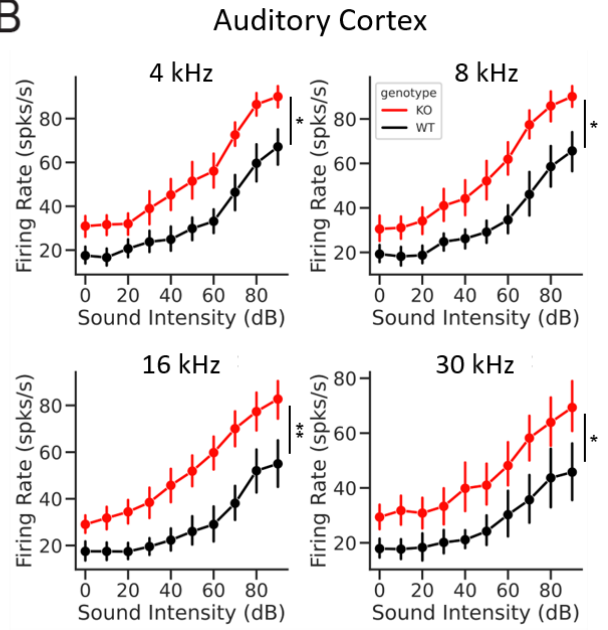

Supplement: S2 Fig — (A) Multi-unit spiking activity recorded from the inferior colliculus of wildtype (WT, black) and Fmr1 KO (red) littermates in response to individual frequencies (4, 8, 16, and 30 kHz) across intensities (0–90 dB SPL, 10 dB steps). No significant genotype difference was observed at any frequency (GLLM: 4 kHz: p = 0.402, 8 kHz: p = 0.577, 16 kHz: p = 0.248, 32 kHz: p = 0.843). (B) Multi-unit spiking activity recorded from the auditory cortex of wildtype (WT, black) and Fmr1 KO (red) littermates in response to individual frequencies (4, 8, 16, and 30 kHz) across intensities (0–90 dB SPL, 10 dB steps). Significant genotype differences were observed at each individual frequency (GLMM: 4 kHz: *p = 0.012, 8 kHz: *p = 0.014, 16 kHz: **p = 0.001, 32 kHz: *p = 0.024). All values are means ± SEM. Data and code underlying this figure can be found at http://doi.org/10.5281/zenodo.15559344. (PDF) [file pbio.3003248.s002.pdf]

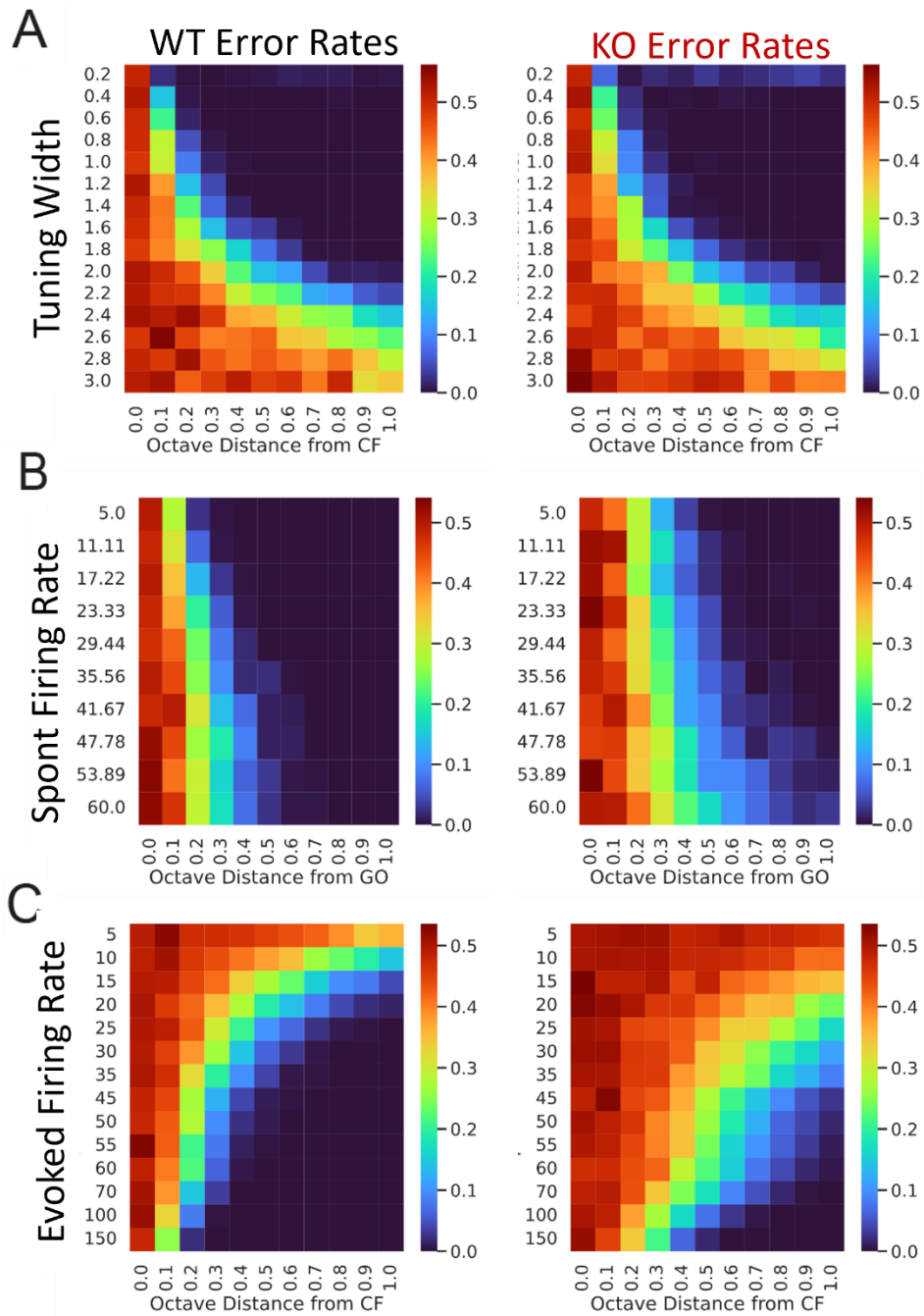

Supplement: S3 Fig — (A) Heatmaps for error rate as a function of systematically varying auditory cortex (ACx) tuning width for the wildtype (WT, left) and Fmr1 KO (right) models. Both WT and KO models appear to be equally sensitive to increased tuning bandwidth. (B) Heatmaps for error rate as a function of systematically varying ACx noise parameter (reflecting spontaneous firing rates) for the WT (left) and Fmr1 KO (right) models. The KO model appears to be more sensitive to increases in spontaneous firing rates compared to WT. (C) Heatmaps for error rate as a function of systematically varying ACx amplitude parameter (reflecting peak sound-evoked firing rates) for the WT (left) and Fmr1 KO (right) models. Both models are sensitive to reduction in amplitude, but KO model has consistently poorer accuracy at all amplitude levels. (PDF) [file pbio.3003248.s003.pdf]

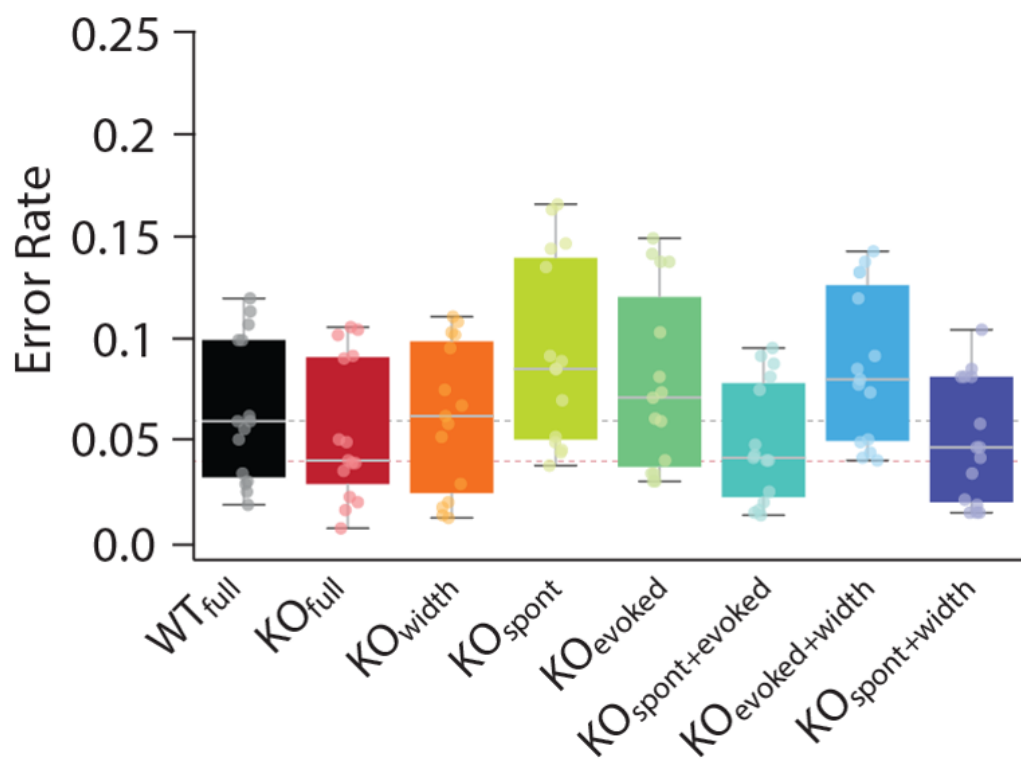

Supplement: S4 Fig — Decoder performance for No-Go tones 1/3–2/3 octave from Go tone as a function of model parameters using physiological data from inferior colliculus (IC) of wildtype (WT) and Fmr1 KO (KO) animals. Error rate was determined for models using all WT (WTfull) or KO (KOfull) parameters, as well as for each unique combination of individual KO parameters for spontaneous firing rates (spont), peak sound-evoked firing rate (evoked), and tuning width (width). Gray and pink dashed lines represent median WTfull and KOfull error rates. While there was a significant effect of model parameter on decoder performance (Kruskal–Wallis Test, *p < 0.0179), there was no difference in performance between any KO model permutation compared to WTfull (Dunn’s test, p = 1.0). Box plots represent the median, 25th, and 75th percentiles. Whiskers represent the minimum and maximum values except for outliers. Boxplots dots represent separate model runs (10,000 repeats each). Source data for this figure are in S1 Data, S4 sheet. (PDF) [file pbio.3003248.s004.pdf]
